# Modeling of Heat Treatment Processes in a Vortex Layer of Dispersed Materials

**DOI:** 10.3390/ma18235459

**Published:** 2025-12-03

**Authors:** Hanna Koshlak, Anatoliy Pavlenko, Borys Basok, Janusz Telega

**Affiliations:** 1 Faculty of Environmental Engineering, Geodesy, and Renewable Energy, Kielce University of Technology, Aleja Tysiąclecia Państwa Polskiego, 7, 25-314 Kielce, Poland; apavlenko@tu.kielce.pl; 2 Department of Electricity Supply, National Technical University of Ukraine “Igor Sikorsky Kyiv Polytechnic Institute”, Borschagivska Str., 115, 03056 Kyiv, Ukraine; borys.basok@gmail.com; 3 Institute of Engineering Thermophysics of the National Academy of Sciences of Ukraine, Marii Kapnist, 2a, 03057 Kyiv, Ukraine; 4 Institute of Fluid-Flow Machinery Polish Academy of Sciences, Generała Józefa Fiszera 14, 80-231 Gdańsk, Poland

**Keywords:** porous fly ash composites, vortex-layer reactor, numerical process modeling, heat and mass transfer, thermal insulation materials, porous media engineering, structure-property relationships, sustainability

## Abstract

Sustainable materials engineering necessitates the valorization of industrial by-products, such as coal fly ash, into functional, high-performance materials. This research addresses a core challenge in materials synthesis: establishing a deterministic technology for controlled porous structure formation to optimize the thermophysical properties of lightweight thermal insulation composites. The primary objective was to investigate the structural evolution kinetics during the high-intensity thermal processing of fly ash-based precursors to facilitate precise property regulation. We developed a novel, integrated process, underpinned by mathematical modeling of simultaneous bloating and non-equilibrium heat transfer, to evaluate key operational parameters within a vortex-layer reactor (VLR). This framework enables the a priori prediction of structural outcomes. The synthesized composite granules were subjected to comprehensive characterization, quantifying apparent density, total porosity, static compressive strength, and effective thermal conductivity. The developed models and VLR technology successfully identified critical thermal exposure windows and heat flux intensities of the heating medium required for the reproducible regulation of the composite’s porous architecture. This precise structure process control yielded materials exhibiting an optimal balance between low density (<400 kg/m^3^) and adequate mechanical integrity (>1.0 MPa). This work validates a scalable, energy-efficient production technology for fly ash-derived porous media. The established capability for predictive control over microstructural development provides a robust engineering solution for producing porous materials, significantly contributing to waste reduction and sustainable building practices.

## 1. Introduction

Porous materials (PMs) are defined by a specific structure that determines their properties and functional appeal across various technological processes, notably in thermal insulation. The relationship between material structure and its resulting properties is a central focus of extensive research.

For instance, several authors [1,2] have generated neural network-based models of porous media for machine learning applications, specifically to predict the mechanical properties of these materials. The reported findings suggest that the mechanical properties predicted by the network are analogous to the actual, measured values. Furthermore, modeling results [3,4] have demonstrated that beyond pore size, the distribution and shape of pores are also crucial determinants of a material’s mechanical performance.

In general, a significant body of scientific literature is available, dedicated to investigating the functional dependence of material properties on their porosity. This extensive research base opens up considerable opportunities for predicting properties during the PM manufacturing stage. Significantly, methods based on computational or numerical experiments provide an effective means for assessing this dependency [5,6]. For example, the authors of a recent review article [7] furnished a comprehensive analysis of current advancements in predicting the properties and applications of porous materials, whilst also highlighting key trends in their utilization.

A crucial, albeit more complex, area of investigation is the functional dependency of material thermal conductivity on their porous structure [8,9]. Studies into such correlations are vital for various applications, including thermal insulation technologies [10,11], PMs utilized in solar energy [8,12], and in the construction industry [13,14].

It is generally accepted [15,16] that the primary factors influencing thermal conductivity are moisture content, temperature difference, and bulk density/porosity. While various publications have summarized this relationship, the effective thermal conductivity of materials is also fundamentally determined by structural parameters [17,18]. Specifically, refs. [19,20] conducted a comprehensive assessment of how the shape, distribution, and overlap of the porous structure affect thermal conductivity. Their findings established that pores possessing the largest contact area, irrespective of the porosity type, significantly influence the functional properties of the materials.

Further research [21,22] has proposed novel models for predicting the effective thermal conductivity of porous media, aiming to quantitatively assess the influence of cell size on this property. These studies demonstrated that non-uniformity in cell size increases the effective thermal conductivity. Similar conclusions were reached by the authors of [23] in their investigation of open porosity. Conversely, refs. [24,25] concluded that pore shape and overlap exert a key influence on the reduction in thermal conductivity; however, this coupled effect has not yet been studied quantitatively.

More recently, ref. [26] proposed various three-dimensional models to analyze heat transfer within PMs. Intriguingly, this work established that pore overlap reduces the thermal insulation characteristics, regardless of pore shape, a finding that appears to contradict data presented in previous studies. The practical implementation of these structural findings is exemplified in the work of [27]. The authors investigated a semi-crystalline structure formed through the reaction between a solid aluminosilicate and an activation solution as a method for developing a matrix with the required structural parameters. Thus, this body of work establishes a clear link between a material’s thermophysical and mechanical properties and its structural characteristics. However, this raises the fundamental question: how can the process of porous matrix formation be made controllable? The findings in [27] suggest that the controllable formation of a porous matrix can be achieved through two primary approaches: the selection of raw material components and the optimization of technological regimes. Critically, the presence of aluminosilicates in the raw mixture allows for the integration of both these methods. As these compounds are widely available in fly ash, this explains the substantial number of publications dedicated to technologies for forming PMs based on ash mixtures [28,29]. Research into the production of geopolymer materials is broadly diverse. For example, studies in [30,31,32] used fly ash as the primary raw material, with the main differentiation in these investigations being the method used to adjust the geopolymer’s chemical composition—typically via the introduction of additives that alter the Si:Al or Si:Na ratio. The utilization of coal fly ash (CFA) as a core component in synthesizing a porous structure, coupled with the optimization of pore-forming technological regimes, may be considered a key direction for further research [33,34,35,36]. Nonetheless, currently, there appears to be a notable absence of publications specifically dedicated to these combined investigations.

Among the simplest methods for the controllable formation of a porous structure are the sol–gel techniques. These are typically achieved through acid leaching or the addition of CO_2_ [37,38,39]. This approach is considered not only more economical but also environmentally benign and, consequently, more promising. These sol–gel technologies could potentially be integrated with structural prediction methods involving the numerical simulation of porous matrices exhibiting diverse morphologies [40,41]. If a known correlation between the technological regimes and the structural and thermophysical characteristics of the PMs is established, their synthesis could thus be rendered fully controllable.

Various synthesis strategies have been developed for the design of porous structures [42,43,44], which simultaneously define the existing challenges and promising avenues for controllable pore formation and the functionalization of porous engineering. These strategies predominantly involve applying different combinations of initial mixture composition prior to the pore-forming process.

## 2. Materials and Methods

### 2.1. Raw Materials and Characterization

The study utilized coal fly ash (CFA) as the principal technogenic component, sourced from a local thermal power plant in Poland. The CFA was classified as class F according to ASTM C618 [45]. Ordinary portland cement (OPC), specifically CEM I 42.5 R, was incorporated as the primary binding agent. The detailed chemical composition of the CFA, determined via X-ray Fluorescence (XRF) analysis (Philips, PW 2404, Magic Pro, Amsterdam, The Netherlands), is presented in Table 1. The high content of SiO_2_ and Al_2_O_3_ (>70%) confirms the class F designation and its suitability for alkaline activation. The material exhibits a slightly alkaline pH of 11.75.

**Figure 1:**
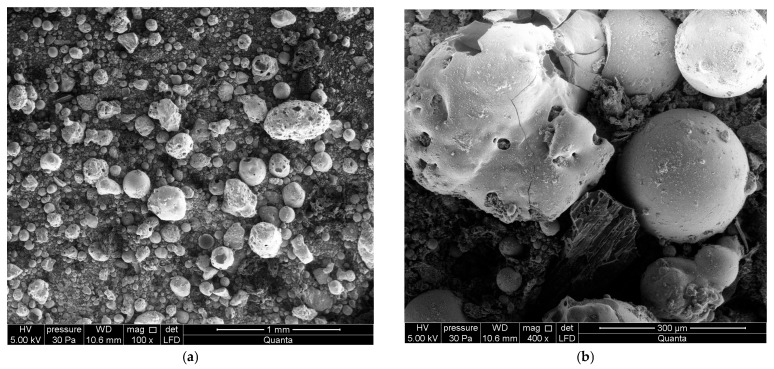
Morphology of the raw CFA: (**a**) Low-magnification SEM image showing particle size distribution; (**b**) High-magnification image of typical spherical glassy cenospheres and plerospheres.

Crucially, the morphology and microstructural characteristics of the raw CFA particles were meticulously examined using scanning electron microscopy (SEM), model QUANTA FEG 250 (Amsterdam, The Netherlands) and a Nikon Eclipse Ci-LED microscope (Tokyo, Japan). The SEM analysis confirmed the typical heterogeneous nature of the fly ash, primarily comprising spherical, glassy cenospheres and plerospheres with varying diameters, as visually represented in Figure 1.

This spherical morphology is known to be beneficial for enhancing the flowability of the raw mixture and acting as effective pore formers during the bloating process. Distilled water was used for mixing all formulations.

The granulometric characteristic of the raw coal fly ash is a fundamental material property, essential for controlling the reproducibility and homogeneity of the precursor mixture. The fineness of the particles directly influences the specific surface area, which governs the dissolution rate and subsequent stabilization of the blend. To quantify these characteristics, a detailed sieve analysis was executed. A 1 kg sample of the raw CFA was subjected to 5 min of agitation using an electromechanical sieve shaker with mesh apertures of 63 mµ, 125 mµ, and 180 mµ. The results confirm a predominantly fine material profile: the majority of the material mass (63.8% wt) resides within the 63–125 mµ intermediate range. The fraction below 63 mµ accounts for 35.3% wt, while the coarse fraction (particles exceeding 180 mµ) constitutes a marginal 0.5% wt. Full details of this particle size distribution are comprehensively documented in our previous work [46], validating the high surface area necessary for optimal reaction kinetics and the desired initial consistency of the precursor blend.

### 2.2. Principles of Foaming and Structural Formation

In the manufacturing of PMs based on hydrosilicates, particularly within low foaming temperature ranges, the expansive agents can include hydrated water and other gases released during chemical reactions. Gas-evolution foaming relies on the gas generated during the porous structure formation process remaining entrapped within the mass, causing it to expand and create the desired cellular structure. Upon heating the hydrosilicate, a mineral melt is initially formed, and its viscosity progressively decreases. Concurrently, the gaseous phase begins to grow, resulting in a proportional increase in its internal pressure. A critical point is subsequently reached when the pressure exerted by the gaseous phase (water vapor) surpasses the combined forces of viscous resistance and surface tension, which then triggers the foaming of the melt. The optimal conditions for effective foaming involve elevated medium viscosity often achieved through the formation of low-temperature eutectic melts combined with a reduced surface tension of the softened glass, a state fundamentally governed by the material’s chemical composition and temperature. Therefore, for successful expansion to take place, the raw material must be in a pyroplastic state during the entire dehydration process. Specifically, at a given moisture content, the initial dehydration temperature must exceed the material’s setting (or solidification) temperature, and this favorable relationship must be maintained until complete dehydration is achieved.

The formation of the cellular structure occurs in three distinct stages: bubble nucleation, growth, and finally, stabilization of the bubble’s size and location. Gas bubbles typically nucleate within the pyroplastic melt, specifically on the active Si-OH sites present on the surface of the silicon. The subsequent growth of the gas bubble, leading to pore formation, is a complex process governed by the rheological properties of the melt, namely, its viscosity and surface tension, both of which can be controlled by the foaming temperature regimes. Pore formation is influenced by a range of physical and physicochemical processes. The initial physical process involves the softening of the hydrosilicate raw material particles due to temperature increase, which correspondingly raises the material’s viscosity and facilitates the optimal foaming regime. Subsequently, as the temperature gradually increases, the gaseous phase grows through chemical reactions, driven by the evaporation of bound water or the application of an additional foaming agent. Gas bubble nucleation and pore formation occur simultaneously throughout the entire mass. Vapor bubbles nucleate at the active Si-OH sites, gradually grow, and merge with other bubbles if the distance between nucleation points is less than the bubble’s diameter. However, the continued growth of these bubbles is not unbounded. The cessation of bubble growth is regulated by the rheological parameters of the mass: specifically, the yield stress and viscosity. This phenomenon is explained by the fact that the merging of bubbles is accompanied by a decrease in internal bubble pressure, which slows down the growth rate. Consequently, despite variations in the intensity of bubble formation, the resulting porous structure will tend towards homogeneity. In summary, the process of porous structure formation encompasses the nucleation, creation, and growth of gas bubbles. The intensity with which these phenomena occur is determined by the intensity of heat supply and the uniformity of heat distribution within the raw material particle. Furthermore, it is critically influenced by the kinetics of the chemical reactions among the various substances present in the raw mixture.

### 2.3. Formulation Strategy and Component Modification

A critical review of initial mixture compositions and established methods for synthesizing PMs indicates a significant drawback in the extensive introduction of gelatinising agents. Such agents are known to disrupt the structure of silicate compounds, leading to the formation of silicic acid hydrosilicate gel, which consequently retains less water. Our research adopts a similar foundational strategy to existing works but uniquely relies on the application of technogenic components predominantly contained within CFA and OPC.

The synthesis process of PMs is further complicated at relatively low temperatures by the sluggish internal heating of the raw mass. This heat transfer bottleneck results in substantial non-uniformity of the pore structure. Consequently, a vital prerequisite for producing PMs with desired properties is the meticulous synchronization of the crystallization rate with the chemically bound water removal rate during pore formation.

In existing literature, the initial stage of porous structure formation typically involves preparing a plastic composition that is subsequently subjected to thermal treatment at various temperatures. The resulting porosity type and pore dimensions are directly dependent on the applied temperature. Generally, these studies primarily focus on identifying the optimal blend of raw materials. Our research adopts a similar foundational strategy, but we uniquely rely on the application of technogenic components predominantly contained within CFA and OPC. Thus, our method for PM production is distinguished by its mixture composition, initial volume content, consistency, and the specific technique for porous structure formation. Detailed accounts of this method are presented in our previously published works [47,48].

The novelty of the current investigation lies in providing a solution to the problem of optimal thermal treatment. This involves precisely coordinating the evaporation (pore formation) processes with the material hardening duration to enable the a priori prediction of the average pore size and overall porosity in the synthesized product.

### 2.4. Raw Mixture Composition and Preparation

The composition of the raw mixture based on CFA for the investigated ranges of mass fractions is presented in Table 2.

To investigate the influence of OPC content on the optimal thermal response and final structure, three distinct raw mixtures were prepared. The compositions, detailed in Table 3, represent a controlled variation in the primary binding agent (OPC) against the technogenic filler (CFA).

The dry components were blended for 5 min using a laboratory-scale planetary mixer (ARE-250 CE, THINKY Corp., Tokyo, Japan). A measured volume of water was then added to achieve a pseudo-plastic consistency (defined by a water-to-solids ratio of 0.35 to 0.40), which is optimal for the subsequent granulation and thermal processing stages. The wet mixture was then granulated to form raw granules with a target average diameter of 2.5 ± 0.5 mm.

**Figure 2:**
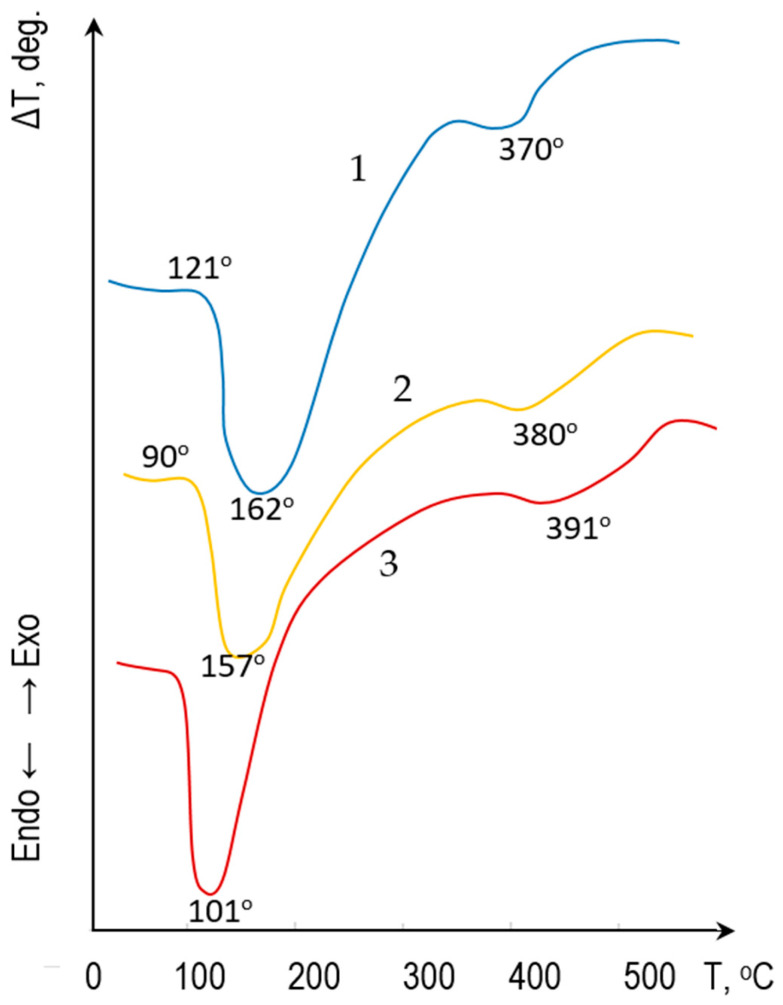
DTA of the raw mixture with varying compositions: 1—80 mass parts CFA and 5 mass parts OPC; 2—70 mass parts CFA and 15 mass parts OPC; 3—50 mass parts CFA and 30 mass parts OPC.

The thermal behavior of the fly ash-cement mixture was assessed using differential thermal analysis (DTA) coupled with thermogravimetry (TG). The DTA/TG was conducted under an inert atmosphere (nitrogen) using a NETZSCH STA 449 F3 Jupiter analyser (Selb, Germany) at a heating rate of 10 °C/min up to 1000 °C. The DTA curves (Figure 2) registered four distinct endothermic effects accompanied by mass loss. The first endothermic effect, observed in the 70–200 °C range, is attributed to the removal of adsorbed water from the raw gel-like material and crystallization water from the calcium hydrosulfoaluminate (AFt) phase. The second endothermic effect, occurring at 370–380 °C, corresponds to the dehydration of calcium hydroxide Ca(OH)_2_, a key hydration product of the OPC.

### 2.5. Thermal Processing Stages

The determining factor for the controlled formation of the PM was the heating rate of the mixture. The optimization of the thermal treatment regime was performed based on empirical studies of the thermal bloating processes and material property changes. The controlled processing technology involves three critical stages, which define the key thermal and physical transformations, as detailed in Table 4.

The intensive thermal treatment during stage II and stage III is proposed to be performed within a vortex-layer reactor. The high intensity of heat and mass transfer processes achieved in VLR technology is sufficient to optimize the timing of crystallization and the formation of the PM porous space, ensuring a fixed final structure.

### 2.6. PM Characteristics and Pore Analysis

The determination of pore sizes in both materials was performed using a combination of micro- and macro-photography of the sample surfaces, followed by digital image processing in a dedicated graphics editor. For macro-photography preparation, several transverse cross-sections, sized 20 × 50 × 50 mm, were prepared. From these images, a dataset of 200 pore size values was sampled and analyzed. Microphotographs were acquired using a Nikon Eclipse Ci-LED microscope.

The thermal conductivity (λ)  measurements were performed using an HFM 436/3 Lambda heat flow meter apparatus (NETZSCH-Gerätebau GmbH, Selb, Germany). Accurate thermal conductivity determination for porous materials with rough surfaces presents challenges due to the difficulty in preparing perfectly flat and parallel sample surfaces. Such surface imperfections can lead to significant thermal contact resistance in the air gaps between the instrument’s plates and the sample surfaces. If this contact resistance becomes substantial, particularly with larger pore sizes, the temperature sensors embedded in the instrument’s plates may not accurately reflect the true temperature difference across the sample itself. To address this, additional thermocouples of small diameter were directly embedded onto the surfaces of the samples. For this study, three pairs of samples (each 305 mm × 305 mm with a thickness of 50 mm) of both the developed porous material and foamed concrete were prepared and placed within the apparatus. The sample thermocouples were connected to the data acquisition channels typically used for the instrument’s plate thermocouple. Subsequently, the automatic offset control feature within the instrument’s software dynamically adjusted the plate temperatures during the test to maintain the desired temperature difference precisely across the sample. Equilibrium parameters were set to a strict tolerance of 0.1%. During the measurements, the temperature difference between the two HFM plates was approximately 18 K, while the actual temperature difference across the sample itself was maintained at 7 K. The reliability of the obtained data was assessed through statistical analysis. For the relationship between thermal conductivity and density, a standard error of *S* = 0.018 and a correlation coefficient *r* = 0.996 were observed. Similarly, for the dependence on the average pore radius (*R*), the standard error was 0.021, with a correlation coefficient *r =* 0.994. These statistical indicators confirm the high accuracy and strong correlation of the measured and modeled thermal conductivity data.

The apparent porosity and water absorption capacity of the synthesized PMs were determined using the standard water saturation method, adhering to relevant international standards (ASTM C373—“Standard test method for water absorption, bulk density, apparent porosity, and apparent specific gravity of fired whiteware products”) [49]. For each batch of material, three representative samples (n = 3), typically with dimensions of approximately 20 × 20 × 20 mm (for granulated samples, representative collections approximating this volume were used), were prepared. Samples were dried in a laboratory muffle furnace NT BIG 20 K (Poland) at 105 ± 5 °C for 24 h. The dry mass of each sample was then measured to the nearest 0.001 g using an analytical balance (Mettler Toledo MS204TS, Columbus, OH, USA).

## 3. Mathematical Modeling of Pore Formation Dynamics

**Figure 3:**
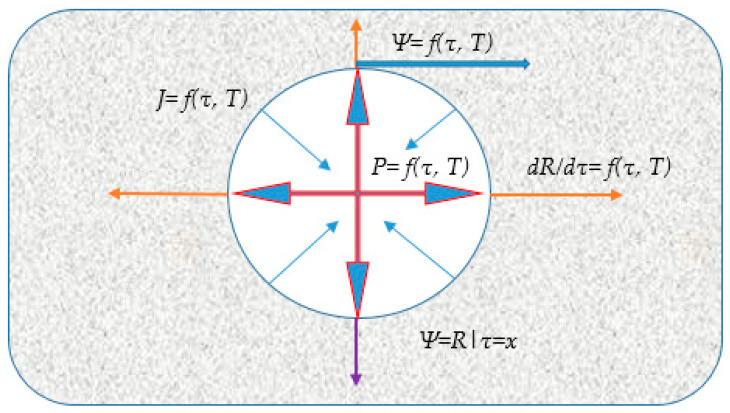
Schematic diagram of the vapor-gas pore growth model: *P* = *f*(*T*, *τ*)—the pressure exerted by the expanding vapor/gas inside the pore (red arrows); Ψ=f(*T*, *τ*)—viscous resistance exerted by the surrounding pyroplastic mass, acting against pore expansion (blue arrows); J= *f*(*T*, *τ*)—the mass flux (thin blue arrow); $\frac{dR}{d\tau }=f(T,\;\tau )$—the rate of pore radius growth (orange arrows), $\Psi =R\left|\tau =x\right.$—stabilization condition (purple arrow).

The foaming process must be precisely regulated so that, at the stage of intense gas evolution, the mixture exhibits properties optimal for pore nucleation and growth. Crucially, towards the completion of gas evolution and porous structure formation, the resultant structure must be fixed and stabilized by a relatively rapid increase in the mixture’s viscosity and yield stress. Structural outcomes are highly sensitive to the kinetic balance. If the rate of vapourisation into the pores exceeds the rate of mass viscosity increase, the material will yield an open porous structure with a chaotic pore distribution upon final expansion. Conversely, if the rate of viscosity increase surpasses the rate of gas evolution, the final porosity achieved will be insufficient. Therefore, only the purposeful, targeted control of these competing processes allows for the reliable synthesis of a material with the desired porosity. A schematic representation of the pore formation process is provided in Figure 3.

The formation of the porous structure is a dynamic process, meaning all modeled parameters are subject to change over time. Consequently, the developed model must incorporate an equation for the quantitative evaluation of the foaming process. This is achieved by imposing a condition where all structural changes within the mixture are fixed at a precisely defined moment in time, τ=x. At this instant, key variables such as the yield stress $\left(\Psi \right),the\;rate\;of\;pore\;radius\;growth\;R\left(\frac{dR}{d\tau }\right),$ the mass flux J, and the internal gas pressure *P* can be treated as constant.

To accurately calculate the pressure within the gas pore, all parameter values must be known for this specific time instant, τ=x. At this point, the gas pressure inside the pore must reach a certain equilibrium value $P_{ecv}$, which is determined by the yield stress Ψ. However, as the mixture foams, the internal gas pressure P will continuously decrease, and the foaming process will immediately cease once this pressure reaches $P_{ecv}$. It is important to note that, at the conclusion of the mixture’s foaming process, the internal gas pressure still exceeds the atmospheric pressure. The developed mathematical model is ultimately designed to determine the final stable pore size based on the predetermined thermal treatment temperatures.

### 3.1. Modeling the Dynamics of Pore Growth

As demonstrated in the Introduction, pore size is a fundamental parameter governing the thermophysical properties of the final PM. Therefore, achieving a material with a specific mean pore diameter and predictable porosity allows for the a priori assumption of its thermal conductivity. The resulting pore size is directly defined by the energetic parameters of the foaming technology. We assume that the foaming of the raw material mixture, comprising the fly ash-cement gel, is driven by the formation and subsequent growth of a steam phase during the intensive heating of the wet mixture (stages 2 and 3 of the process).

The dynamic characteristic that determines the direction and magnitude of the change in the steam pore size is the difference in stresses induced by the pressure within the vapor region and the opposing resistance from the pore’s boundary surface.

The dynamics of growth or contraction of a steam pore within the semi-solidifying gel medium are characterized by the modified Rayleigh–Plesset equation [50]. The form presented below focuses on the acceleration of the pore boundary $\frac{dw}{d\tau }$, defined as the rate of change in the pore growth speed *w* over time *τ*. The equation used to model the acceleration of the pore boundary is:

(1) $$\frac{dw}{d\tau }=-\frac{1.5\rho_{liq}w^{2}+P_{sur}-P_{g}}{\rho_{liq}R}=-\frac{1.5\rho_{liq}w^{2}}{\rho_{liq}R}+\frac{P_{g}\left(T\right)-P_{sur}}{\rho_{liq}R},$$

where $P_{g}$ is the total internal pressure of the vapor-gas mixture inside the pore, Pa; $P_{sur}\;$ is the external pressure exerted by the surrounding gel/liquid, Pa; $\rho_{liq}$ is the density of the liquid/gel mixture, $\frac{\mathrm{kg}}{m^{3}}$; w is the pore growth speed, $\frac{m}{s}$; R is pore radius, m; τ is time, s.

The dynamics of pore expansion are solved by integrating the modified Rayleigh–Plesset equation for $\frac{dw}{d\tau }$ (1) under three distinct thermodynamic conditions, primarily governed by the relationship between the internal vapor pressure, $P_{g}\left(T\right)$, and the external pressure, $P_{sur}$. When the internal vapor pressure dominates, $\frac{P_{g}\left(T\right)-P_{sur}}{1.5\rho_{liq}}>0,$ the pore accelerates towards its maximum velocity. When the external pressure dominates, $\frac{P_{g}\left(T\right)-P_{sur}}{1.5\rho_{liq}}<0,$ the pore velocity decreases, leading to potential contraction. When the net driving pressure is zero, $\frac{P_{g}\left(T\right)-P_{sur}}{1.5\rho_{liq}}=0,$ the pore growth velocity w reaches an immediate, steady state.

These solutions are the result of integrating $\frac{dw}{A-Bw^{2}}=-\frac{1.5\rho_{liq}}{\rho_{liq}R}d\tau$:

(2) $$\left\{\begin{array}{l} \;\;\frac{P_{g}\left(T\right)-P_{sur}}{1.5\rho_{liq}}>0,\;\;\frac{1}{2\sqrt{A}}\ln\left|\frac{w-\sqrt{A}}{w+\sqrt{A}}\right|=-\frac{1.5\rho_{liq}\tau }{\rho_{liq}R}+C\;\\ \frac{P_{g}\left(T\right)-P_{sur}}{1.5\rho }<0,\;\;\;\sqrt{B\;\;}arctan(w\sqrt{B})=-\frac{1.5\rho_{liq}\tau }{\rho_{liq}R}+C,\\ \;\;\;\;\frac{P_{g}\left(T\right)-P_{sur}}{1.5\rho_{liq}}=0,\;\;\;\frac{1}{w}=-\frac{1.5\rho_{liq}\tau }{\rho_{liq}R}+C\; \end{array}\right.$$

where $A=\frac{P_{g}\left(T\right)-P_{sur}}{1.5\rho_{liq}}$ is growth constant, $B=\frac{1.5\rho_{liq}}{P_{sur}-P_{g}\left(T\right)}$ is contraction constant, *C* is the integration constant determined by initial conditions.

Solving the interim solutions (2) for the instantaneous velocity *w* and applying the initial condition w = w_0_ at *τ* = 0, where α is defined as $\alpha =\sqrt{\frac{P_{g}\left(T\right)-P_{sur}}{1.5\rho_{liq}}}$:

(3) $$w\left(\tau \right)=\left\{\begin{array}{c} \frac{\alpha \left(w_{0}-\alpha \right)e^{\frac{1.5\rho_{liq}\tau }{\rho_{liq}R}}+w_{0}+\alpha }{\left(w_{0}+\alpha \right)-\left(w_{0}-\alpha \right)e^{\frac{1.5\rho_{liq}\tau }{\rho_{liq}R}}}\cdot \alpha ,\;if\;P_{g}\left(T\right)-P_{sur}>0\\ w_{0}\frac{\sqrt{\frac{P_{sur}-P_{g}\left(T\right)}{1.5\rho_{liq}}}tan\left(\sqrt{\frac{1.5\rho_{liq}}{P_{sur}-P_{g}\left(T\right)}}\frac{1.5\rho_{liq}\tau }{\rho_{liq}R}\right)}{\sqrt{\frac{P_{sur}-P_{g}\left(T\right)}{1.5\rho_{liq}}}+\frac{1.5\rho_{liq}}{w_{0}}tan\left(\sqrt{\frac{1.5\rho_{liq}}{P_{sur}-P_{g}\left(T\right)}}\frac{1.5\rho_{liq}\tau }{\rho_{liq}R}\right)},\;if\;P_{g}\left(T\right)-P_{sur}<0\\ \frac{w_{0}\rho_{liq}R}{1.5\rho_{liq}w_{0}\tau -\rho_{liq}R},\;if\;P_{g}\left(T\right)-P_{sur}=0 \end{array}\right.,$$

where $w_{0}$ is the initial pore growth speed (at *τ* = 0).

Focusing on the third case, where the net driving pressure is zero ${(P}_{g}\left(T\right)-P_{sur}=0)$, the simplified equation describes the velocity during the final stabilization phase:

(4) $$w=\frac{w_{0}\rho_{liq}R}{1.5\rho_{liq}w_{0}\tau -\rho_{liq}R}.$$

Equation (4) describes a qualitative characteristic of the foaming process and can be used to predict the change in pore size over time. It highlights that the final pore velocity *w* is determined not only by the pore size *R* and mixture density $\rho_{liq},\;$ but also by the initial velocity *w*_0_ and, crucially, the duration of the thermal exposure τ.

### 3.2. Thermodynamic Criteria for Gas-Forming and Expansion Processes

The mechanisms by which mineral fillers and chemical reagents influence the foaming processes can be determined by analyzing the combined equation of the first and second laws of thermodynamics. This analysis uses the change in the Gibbs free energy ∆G to predict the intensity and direction of change in the system’s energetic state.

The general thermodynamic equation describing the change in the system’s energy during pore formation is given as:

(5) dG=dH−TdS+σdF+φdm,

where dG is the change in Gibbs free energy, which forecasts the direction and intensity of the energy change; dH is the enthalpy factor of the system; σ is the surface tension at the pore boundary; dF is the change in the surface area of the pore; dm is the change in the mass of the pore-forming gas; φ is the chemical potential of the pore-forming gas; TdS is the entropy factor.

The change in entropy (*TdS*) occurs as the porous structure forms, driven by the thermal energy absorbed. This change in entropy contributes to the thermodynamic work performed by the system (the raw mixture) as it expands against external pressure and viscous forces to create and enlarge the pores. The Gibbs energy can also be represented as a function of mass, surface area, and chemical potential, separating the bulk material energy from the pore formation energy. The Gibbs energy for the system focusing on the pore-forming gas is:

(6) G=mφ+Fσ,

where *m* is the mass of the pore-former (gas); *F* is the external surface area of the pore nucleus.

The total Gibbs energy of the system, which consists of the energy required for pore nucleus formation and the porous material structure, is:

(7) $$G=G_{mat}+m\varphi +F\sigma ,$$

where $G_{mat}$ is the Gibbs energy of the bulk material.

We consider the equilibrium state of this system, which corresponds to dG=0. If the external pressure $P_{sur}$ equal to the gas pressure in the pore and the temperature T are constant, the equilibrium condition can be written as:

(8) $$TdS=dU+P_{sur}dV,$$

where U is the internal energy and $P_{sur}\;$ is the external pressure (in this case, the pressure within the mixture),

For a gas bubble, the internal pressure $P_{g}$ is related to the external pressure $P_{sur}\;$ and surface tension σ by the Laplace formula:

(9) $$P_{g}-P_{sur}=\frac{2\sigma }{R}.$$

Considering that dG=0, the equilibrium condition leads to the differential of the chemical potential of the pore-forming gas:

(10) $$d\varphi =V_{g,m}dP_{g},$$

where $V_{g,m}$ is the molar volume of the gas (pore-forming agent), $V_{g,m}\approx \frac{RT}{P}.$

Considering that the system’s total mass is constant:

(11) $$m=m_{g}+m_{liq}.$$

(12) $$dG=0\;\Rightarrow dm_{g}\left(\varphi_{g}-\varphi_{liq}\right)+\sigma dF=0.$$

For spherical pores $F=4\pi R^{2}\;$ and $m_{g}=\frac{4}{3}\pi R^{3}\rho_{g}$, leading to the following derivatives:

(13) dF=8πRdR,

(14) $$dm_{g}=4\pi R^{2}\rho_{g}dR.$$

The ratio of these derivatives can be written as:

(15) $$\frac{dG}{dR}=4\pi R^{2}\rho_{g}\left(\varphi_{g}-\varphi_{liq}\right)+8\pi R\sigma ,$$

Under the condition $\frac{dG}{dR}=0$, the term in brackets must be zero:

(16) $$\varphi_{g}-\varphi_{liq}=-\frac{2\sigma }{\rho_{g}R},$$

By substituting the actual values of the pore-forming agent’s chemical potential $\varphi_{g}$ and material’s chemical potential $\varphi_{liq}$, and decomposing them into a series by pressure and radius, the following condition is obtained:

(17) $$\varphi_{g}(P_{g})-\varphi_{liq}(P_{sur})\approx V_{liq,m}(P_{g}-P_{sur}),$$

where $V_{liq,m}\;$ is the molar volume of the surrounding gel/liquid.

From Equation (17), the condition of gas pressure equilibrium in the material during porous structure formation, taking into account the surface tension, is derived:

(18) $$\varphi_{g}=\varphi_{liq}\;$$

After differentiation of the general Gibbs energy Equation (5) by pressure P at constant temperature T=const and considering that ${\left(\frac{\partial G}{\partial P}\right)}_{T}=V,$ the following is obtained:

(19) $${\left(\frac{\partial \varphi }{\partial P}\right)}_{T}=V_{g,m}.$$

Since the liquid phase volume $V_{liq}\;$ is significantly smaller than the gas phase volume $V_{g}$, and the molar volume $V_{g,m}$ of the gas at relatively low pressures can be approximated by the ideal gas law $V_{g,m}\approx \frac{RT}{P}$, the equation simplifies to:

(20) $$\frac{d\ln P}{dT}=\frac{∆H_{vap}}{RT^{2}},$$

where $\;∆H_{vap}$ is the molar enthalpy of vaporization.

By differentiating the equation of equality of potentials (18) by $P_{g}$, and considering the thermodynamic relationships for enthalpy and volume, we obtain:

(21) $$d\ln P_{g}=\frac{∆HdT}{RT^{2}},$$

(22) $$dT_{vap}=\frac{T_{vap}^{2}V_{g,m}}{∆H_{vap}}dP_{g}.$$

Given that the material density $\rho_{liq}$ is almost independent of pressure at low gas pressures, we obtain:

(23) $$\frac{d\rho_{liq}}{dP}\approx 0.$$

Given $T_{vap}=T\;$ and integrating, we finally write the relationship that determines the required process temperature (often referred to as the Clausius-Clapeyron equation):

(24) $$T=T_{vap}=\frac{∆H_{vap}}{∆S_{vap}+R\ln\left(\frac{P_{\infty }}{P_{g}}\right)}.$$

where $∆S_{vap}$ is the molar entropy of vaporization.

This thermodynamic relationship allows for the determination of the heating gas temperature necessary for the material’s thermal treatment:

(25) $$T_{g}=T_{vap},$$

where $T_{g}$ is interpreted as the vapor temperature inside the pore $T_{vap}$, which corresponds to the first endothermic minimum on the DTA curve (Figure 2).

### 3.3. Comprehensive Model for Pore Evolution

Section 3.1 and Section 3.2 established the fundamental hydrodynamic (Rayleigh–Plesset) and thermodynamic (Gibbs free energy) criteria. This section integrates these concepts into a comprehensive system of coupled equations that precisely models the formation and evolution of a steam pore in a viscous, solidifying medium.

The theoretical analysis of gas phase (pore) growth dynamics in the raw mixture during heating relies on the modified Rayleigh–Plesset equation. Modeling the gas phase formation necessitates accounting for the intensity of gas dissolution and the simultaneous liquid phase change, which is governed by the speed of heat and mass transfer processes near the bubble surface. The main factors influencing these processes are the temperature $T_{vap}\;$ and pressure $P_{g}$ of the gas-vapor mixture inside the bubble. Given that the rate of change in the pore size can reach several hundred meters per second, a correct problem formulation must consider a complex of interconnected mechanical and thermodynamic processes occurring in a confined volume at high speed. The overall model is based on the necessity to account for the complex interplay between mechanical forces, heat transfer, and mass exchange occurring at high velocities and in a restricted volume. The model includes the following components:

- model for the kinetics of a gas bubble in a viscous, solidifying liquid.
- model for thermodynamic processes inside the gas-vapor bubble.
- model for heat and mass exchange processes at the bubble boundary.
- modeling of phase transition in the surrounding liquid.
- modeling of heat exchange processes in the liquid surrounding the bubble.

For simplification of the mathematical model, the following assumptions are made:

- the gas-vapor bubble has a spherical shape;
- the liquid is viscous and incompressible.

#### 3.3.1. Hydrodynamics, Mass Transfer, and Equilibrium

The rate of change in the pore radius R is determined by a modification of the Rayleigh–Plesset equation, incorporating viscosity µ, surface tension σ, and the total internal pressure of the vapor-gas mixture $P_{g}$.

The rate of pore growth, $w=\dot{R}$, is determined by integrating the modified Rayleigh–Plesset equation, written here in its acceleration form:

(26) $$\frac{dw}{d\tau }=\frac{d\dot{R}}{d\tau }=\frac{P_{g}-P_{sur}}{\rho_{liq}R}-\frac{1.5}{R}w^{2}-\frac{4\mu_{liq}}{\rho_{liq}R^{2}}.w-\frac{2\sigma }{\rho_{liq}}R^{2},$$

where $P_{g}$ is the total internal pressure of the vapor-gas mixture, Pa; $P_{sur}\;$ is the external pressure exerted by the surrounding gel, Pa; $\rho_{liq}\;$ is the density of the liquid/gel mixture, $\frac{\mathrm{kg}}{m^{3}}$; $\mu_{liq}$ is the dynamic viscosity, Pa ·s; σ is the surface tension coefficient $\frac{N}{m}$; R is the pore radius, m; τ  is time, s.

If evaporation or condensation occurs at the pore surface, the radius R changes due to both radial motion and phase transition:

(27) $$\frac{d\dot{R}}{d\tau }=w+\frac{I_{vap}}{\rho_{liq}},$$

where $I_{vap}$ is the mass flux of the vapor transferred across the unit surface area of the bubble per unit time $\frac{\mathrm{kg}}{m^{2}s}$. The correction for phase transition influence is usually small.

The initial equilibrium state for a pore with radius $R_{0}$ at internal pressure $P_{g0}$ is defined by the Laplace pressure:

(28) $$P_{g0}=P_{sur}+\frac{2\sigma }{R_{0}}.$$

#### 3.3.2. Thermodynamics and Equations of State

The total internal pressure $P_{g}$ changes due to variations in pore size or temperature. According to Dalton’s Law, the total pressure is the sum of the partial pressures of the non-condensable gas $P_{gas}$ and the water vapor $P_{vap}$:

(29) $$P_{g}=P_{gas}+P_{vap}.$$

The Van der Waals equation of state is used to determine the partial pressures of the components:

(30) $$P_{gas}=\frac{R_{\mu }T}{\frac{\mu_{gas}}{\rho_{gas}}-b_{gas}}-\rho_{gas}^{2}\frac{a_{gas}}{M_{gas}^{2}},\;\;P_{vap}=\frac{R_{\mu }T}{\frac{\mu_{vap}}{\rho_{vap}}-b_{vap}}-\rho_{vap}^{2}\frac{a_{vap}}{M_{vap}^{2}},$$

where $R_{\mu }$ is the universal gas constant; T is the temperature of the gas mixture $\left(T_{vap}\right)$; $\mu_{gas}\;$ and $\mu_{vap}$ are the molecular masses; $\rho_{gas}$ and $\rho_{vap}\;$ are the densities; and $a_{i},\;b_{i}$ are the Van der Waals constants. The unknown values here are $T_{vap}$ and the component densities.

The densities of the mixture gases are determined by the influence of mass transfer processes and the change in the bubble radius:

(31) $$\frac{d\rho_{gas}}{d\tau }=\frac{3}{R}\left(I_{gas}-\rho_{gas\;}\frac{dR}{d\tau }\right),\;\;\;\frac{d\rho_{vap}}{d\tau }=\frac{3}{R}\left(I_{vap}-\rho_{vap\;}\frac{dR}{d\tau }\right),$$

where $I_{gas}$ = $I_{g}$ is the mass flux of the non-condensable gas transferred across the boundary $\frac{\mathrm{kg}}{m^{2}s}$.

The First law of thermodynamics is applied to determine the temperature inside the bubble:

(32) $$\frac{dU_{gas}}{d\tau }+\frac{dU_{vap}}{d\tau }=F\cdot q_{wall}-P_{g}\frac{dV}{d\tau },$$

where $q_{wall}$ is the specific heat flux near the bubble surface.

Which can be expanded for a mixture of gases:

(33) $$\frac{d\left(m_{gas}C_{p,gas}+m_{vap}C_{p,vap}\right)T}{d\tau }=4\pi R^{2}q_{wall}-P_{g}\frac{d\left(\frac{4}{3}\pi R^{3}\right)}{d\tau }.$$

Assuming the mass specific heat capacities $C_{p,gas},\;C_{p,vap}$ are constant, differentiation yields the dynamic temperature equation

(34) $$\frac{dT_{vap}}{d\tau }=\frac{3}{R(C_{p,gas}\rho_{gas}+C_{p,vap}\rho_{vap})}\left[q_{wall}-T\left(C_{p,gas}I_{gas}+C_{p,vap}I_{vap}\right)-P_{g}\frac{dR}{d\tau }\right].$$

This equation is coupled to three unknown fluxes: the specific heat flux $q_{wall}$ near the bubble surface, and the mass fluxes $I_{gas}$, $I_{vap}$.

### 3.4. Results of Mathematical Modeling

**Figure 4:**
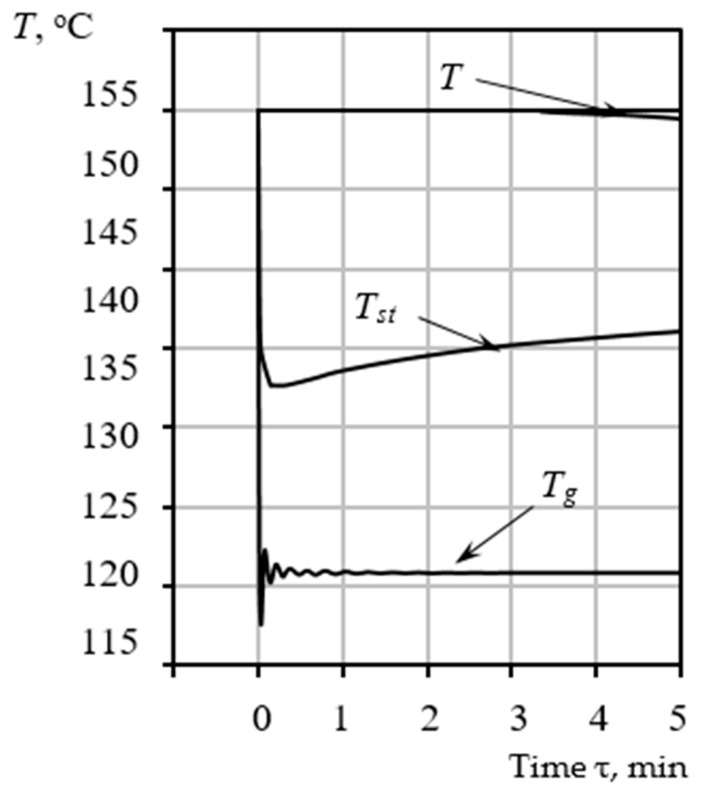
Time-dependent variation in the calculated temperatures: heating gas $T_{g}$, the temperature of the raw mixture $T_{st}$, and pore-forming vapor $T_{vap}$.

**Figure 5:**
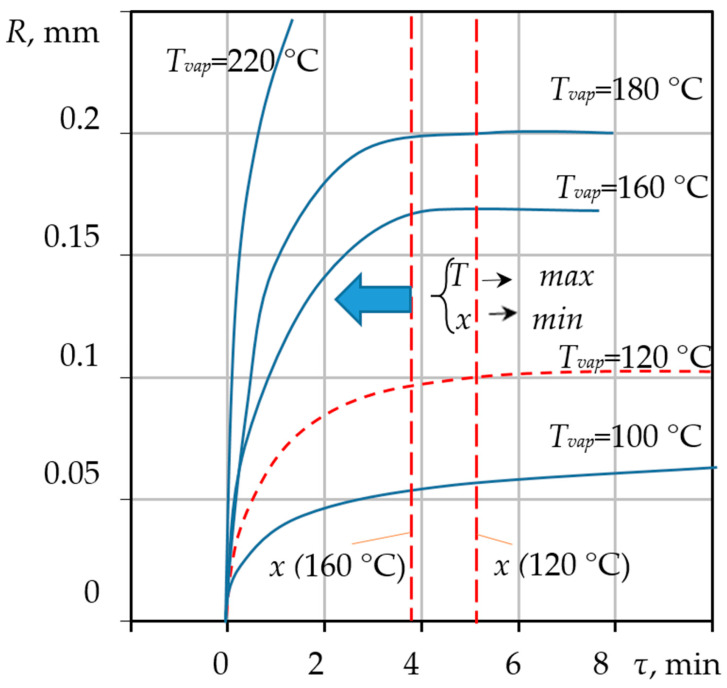
Calculated dynamic change in the average pore radius R as a function of time τ.

**Figure 6:**
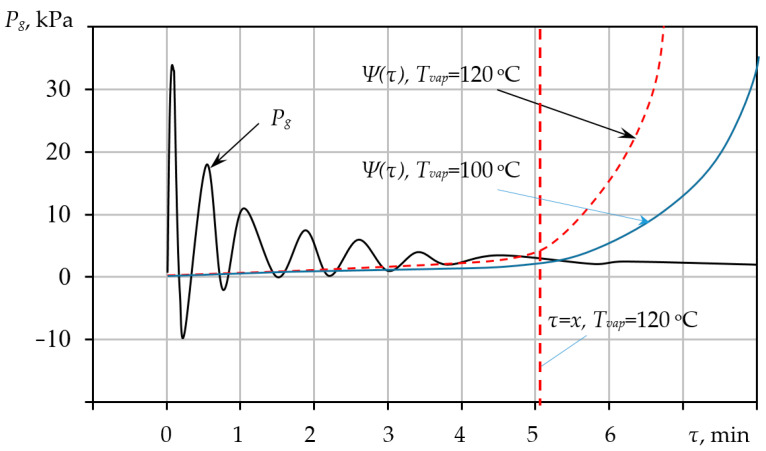
Change in gas pressure $P_{g\;}$ in the pore and yield stress Ψ over time τ.

The results obtained from the numerical solution of the coupled differential equations developed in Section 3 are presented in Figure 4, Figure 5 and Figure 6. These figures illustrate the dynamic changes in key parameters of the pore-forming process over time, providing validation of the comprehensive model. Solving the model equations allows for the deterministic prediction of pore dimensions $R_{p}$, which achieve stabilization upon reaching the equilibrium pressure value $P_{eg}$ within the pore structure. The dynamics of pore radius evolution as a function of the precursor mixture’s heating temperature T provides information regarding the required duration of the thermal treatment $\tau_{res}.$ The model allows us to identify specific temperatures and exposure times necessary to stabilize the desired pore size precisely at the moment when the precursor mixture acquires the necessary rigidity. This material state is defined as a plastic mass ready for subsequent granulation and drying processes within the VLR, which is uniquely capable of ensuring the required high-intensity heat and mass transfer.

As an example for the calculation, the following key parameters were adopted, which are integral to the mathematical model equations: the excess pressure within the mixture $P_{sur}$ = 10^5^ Pa; Ψ = 30 Pa; $\mu_{liq}=0.96\cdot 10^{4}$; $\rho_{liq}$ = 1600 kg/$m^{3}$; $R_{0}$ = 0 m.

#### 3.4.1. Temperature and Pore Growth Dynamics

Figure 4 presents the characteristic trends for three critical temperatures: the temperature of the heating gas $T_{g}$, the temperature of the raw mixture $T_{st}$, and the temperature of the pore-forming vapor inside the bubble $T_{vap}$. The heating gas temperature $T_{g}$ is not an arbitrary input but is determined a priori based on the integrated form of the Clausius-Clapeyron relation (24). This temperature dictates the optimal thermal conditions necessary for controlled synthesis, specifically targeting the required intensity of heat and mass transfer $q_{wall}\;$ to maintain the target $T_{vap}$ while providing the necessary enthalpy ${∆H}_{vap}$ for sustained evaporation.

As evidenced by the graphs (Figure 4), all calculated parameters for the specified conditions stabilize within 5 min. During this brief time interval, the yield stress Ψ must reach a value of 30 Pa to satisfy the stabilization condition $\Psi =P_{ecv}$. Consequently, the pore size growth is fixed at a final radius of R=0.1 mm. If the objective is to achieve larger pore sizes, the temperature of the heating medium must be controlled to ensure a corresponding adjustment in the mixture’s rheological parameters, thereby effectively increasing the time required to reach the equilibrium state.

Figure 5 presents the calculated data characterizing the change in pore radius over time for the processing temperatures indicated in Figure 3 for the calculated conditions.

The simulation results indicate that the pore-forming vapor temperature $T_{vap}$ stabilizes near 120 °C. This specific temperature is of critical importance as it precisely aligns with the first endothermic effect observed during DTA of the raw material. This alignment confirms the initial thermodynamic hypothesis that steam formation is the primary, rate-determining reaction responsible for pore nucleation and initial growth.

By comparing Figure 4 and Figure 5, it is evident that the changes in temperature and the increase in pore size are synchronized in time, and the pore size becomes fixed at a final value of *R =* 0.1 mm. This synchronicity underscores the strong coupling in the system: the energy supplied by the heat flow sustains the phase change (evaporation), which, in turn, drives the hydrodynamic growth of the pore (Rayleigh–Plesset dynamics). The entire process of forming the final porous structure occurs rapidly, typically within 5 min for a 3 mm raw gel particle, confirming the high intensity of the coupled thermal-hydrodynamic phenomena. No further increase in pore dimensions occurs after this stabilization point. Higher or lower processing temperatures correspond to unique values of the final radius *R* and the stabilization time τ=x, both of which are determined using the proposed mathematical model.

Figure 6 illustrates the calculated time evolution of the internal gas pressure $P_{g}$ and the rheological resistance Ψ within the porous mixture, demonstrating the kinetic balance required for structure stabilization. For thermal treatment conducted at a temperature of 120 °C, the duration of pressure stabilization τ  is 5 min. At this precise moment, the mixture solidifies, meaning any further foaming is inhibited and only becomes possible if the vapourisation temperature $T_{vap}\;$ is subsequently increased. The functional dependency of the yield stress Ψ on the time τ  and the vapourisation temperature $T_{vap}$ can be determined for each specific mixture using standard methods, thus enabling the prediction of the final pore size.

#### 3.4.2. Analysis of Mass Transfer Kinetics

**Figure 7:**
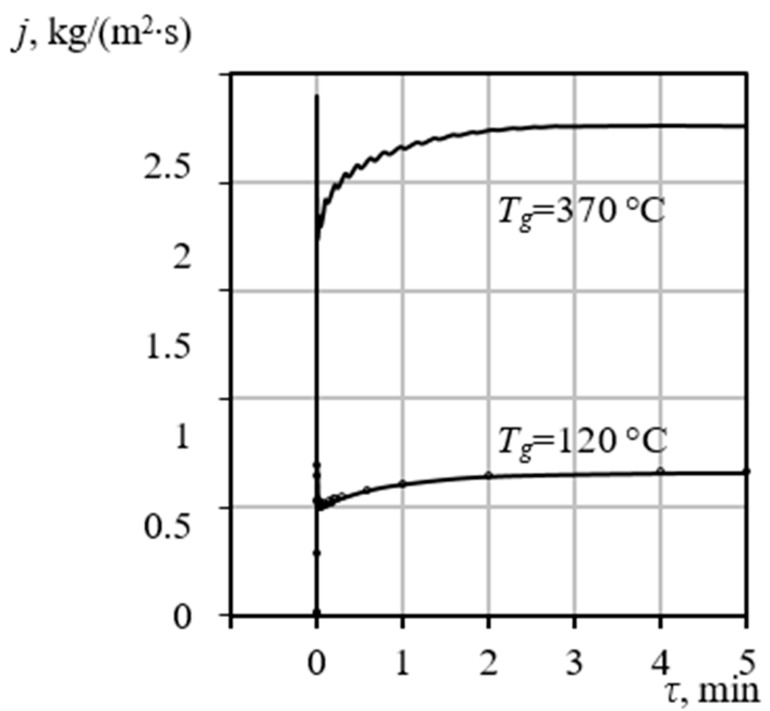
Calculated specific mass flux j across the pore boundary as a function of time τ at two distinct heating temperatures $T_{g}$.

Figure 7 illustrates the specific total mass flux j of the gas-vapor mixture across the bubble surface, defined as $j=I_{vap}+I_{gas}$, as a function of time τ for two distinct heating gas temperatures $T_{g}$. This analysis is crucial for linking external thermal input to the internal pore growth mechanism.

The simulation results reveal two critical kinetic characteristics.

1. The maximum values of the specific mass flux j are consistently observed to correspond with the temperature ranges of the endothermic effects recorded in the DTA data. This correspondence confirms that the peak mass transfer intensity is directly driven by the phase transition (evaporation) occurring at the gel-vapor interface, which requires significant latent heat input.
2. The results strongly demonstrate the dependence of mass transfer kinetics and thus the final porous structure on the external thermal conditions $T_{g}$. At a moderate heating temperature $T_{g}=120\;{}^{\circ}C$, the specific mass flux j stabilizes at a lower equilibrium value, facilitating a slower, more controlled release of vapor. This kinetic regime allows the surrounding gel matrix to potentially solidify or compact around the pore before maximum expansion is achieved, leading predominantly to a structure with closed porosity. Conversely, at a high heating temperature $T_{g}=370\;{}^{\circ}C$, the mass flux increases dramatically, stabilizing around j = 2.6 $\frac{\mathrm{kg}}{m^{2}s}$. This rapid, intense evaporation rate drives a swift and powerful pore expansion that likely exceeds the rate of gel hardening, preventing the formation of dense, closed walls and resulting in the desired open porosity. Crucially, the entire pore formation phase concludes in approximately 5 min. This extremely brief and dynamic processing window establishes a critical technological constraint, confirming the theoretical necessity of employing specialized, high-efficiency equipment, such as VLR, to ensure the uniform and controlled heat application required for precise porosity synthesis.

## 4. Results

### 4.1. Trajectory Dependence on Injection Strategy and Flow Velocity

**Figure A4:**
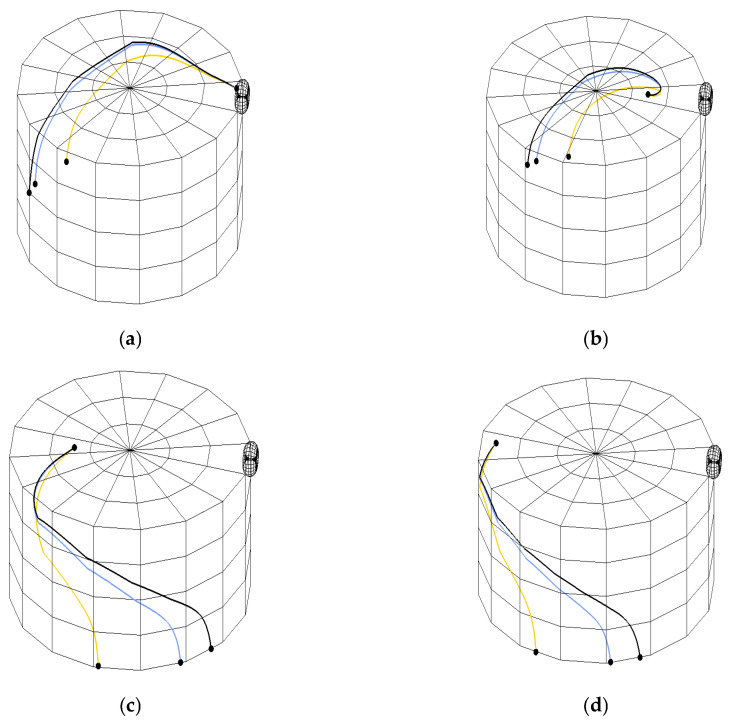
Calculated trajectories of a 3 mm diameter particle over a 5 s period within the VLR: (**a**) axial cross-section; (**b**) detail of axial cross-section; (**c**) radial/tangential cross-section, (**d**) detail of radial/tangential cross-section. The color code for gas velocity $v_{g}\;$ is consistent across all panels: yellow line $v_{g}=5\frac{m}{s};\;$ blue line $v_{g}=10\frac{m}{s},$ black line $v_{g}=15\frac{m}{s}$.

The results derived from the comprehensive two-phase numerical model (encompassing the gas flow analysis in Appendix A.2 and the particle trajectory model in s Appendix A.3 confirm the fundamental working hypothesis: the required particle residence time $\tau_{res}\;$ is a complex and highly controllable function of the gas flow parameters and the particle injection strategy. The analysis of calculated particle trajectories (Figure A4 in Appendix B) revealed critical design and operational dependencies. For a more detailed explanation of the methodology used for the particle trajectory model and the determination of particle suspension limits, please refer to Appendix B.

### 4.2. Technological and Design Implications and Future Directions

The predictive data obtained from this simulation provides a robust foundation for the design optimization and energy efficiency of the vortex reactor. The geometry and length of the calculated trajectories directly determine the requisite equipment size and the corresponding heat transfer medium consumption characteristics. Crucially, the model identifies the particle inlet location and gas flow velocity as the two primary, adjustable control parameters. By precisely tuning these variables, manufacturers can achieve the necessary thermal processing intensity to dictate the final porous structure and target thermophysical properties of the product, minimizing the risk of under- or over-processing.

**Figure 8:**
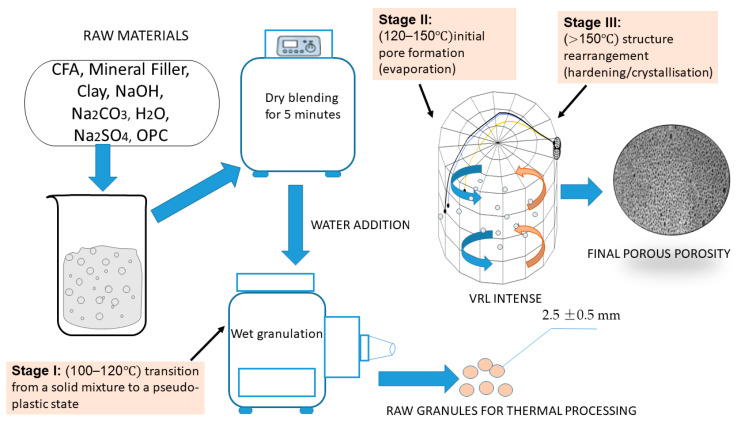
Flowchart of the PM synthesis process. The blue and orange curved arrows indicate the characteristic cyclical, rotating flow pattern (vortex) of the gas and/or the granulated particles within the reactor chamber. The small light circles represent the raw granules (particles) of the fly ash-cement mixture, which are actively suspended and mixed by the gas flow. yellow line $v_{g}=5\frac{m}{s};\;$ blue line $v_{g}=10\frac{m}{s},$ black line $v_{g}=15\frac{m}{s}$.

The experimental investigations performed on a laboratory-scale setup, adhering strictly to the developed methodology, successfully yielded the PM. An overview of the entire synthesis process, from raw material preparation to final thermal treatment, is depicted in Figure 8.

The synthesis of the porous material involves a meticulously controlled, multi-stage process, as illustrated in Figure 8. Initially, the raw components, including CFA, mineral filler, clay, sodium hydroxide, sodium carbonate, water, sodium sulfate, and OPC, are subjected to dry blending in a planetary mixer for 5 min. Subsequently, water is introduced to achieve a pseudo-plastic consistency, characterized by a water-to-solids ratio between 0.35 and 0.40. This mixture is then granulated to form raw granules with an average diameter of 2.5 ± 0.5 mm. Following preparation, the granules undergo a three-stage thermal treatment. Stage I, conducted at 100–120 °C, facilitates the transition from a solid state to a pseudo-plastic paste. Stage II (120–150 °C), performed within a VLR, is critical for the release of free and adsorbed water and the initiation of pore formation through evaporation. Finally, stage III (at temperatures exceeding 150 °C, also in the VLR, completes the process by ceasing major water loss and promoting structure rearrangement, leading to hardening and crystallization. This precisely controlled sequence ensures optimal pore formation and the desired final structure of the PM.

### 4.3. Material Characteristics and Pore Analysis

For comparative analysis, the characteristics of our developed PM were contrasted with those of conventional foamed concrete, which typically exhibits an average pore size of 0.5 mm. To facilitate this comparison, the foaming temperature for our material was specifically set at 130 °C, yielding a material with comparable characteristics for direct evaluation. The final characteristics of this material are detailed in Table 5.

**Figure 9:**
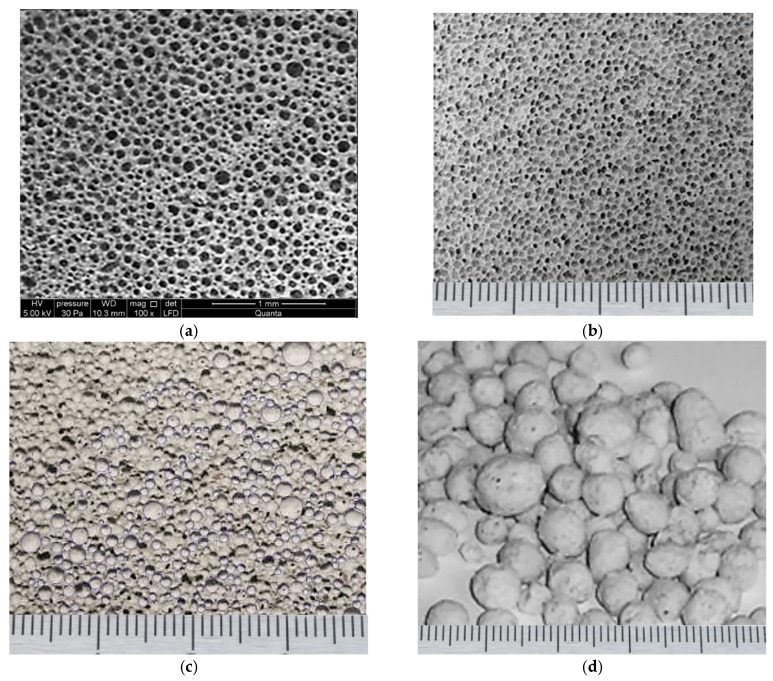
Porous structure of material samples: (**a**) Microstructure of the porous material processed at 120 °C (average pore diameter 0.25 mm); (**b**) Macrostructure of the porous material processed at 130 °C (average pore diameter 0.5 mm); (**c**) Microstructure of reference foamed concrete; (**d**) External view of the synthesized foamed granules.

The resulting the expanded granules is visually presented in Figure 9. The structure of the synthesized material is presented in Figure 9a, providing a visual comparison between the predicted and experimentally obtained pore sizes. Specifically, the material was processed under conditions calculated to yield a pore size of 0.25 mm at a foaming temperature of 120 °C with a process duration of approximately 6 min. The agreement between these predicted and the obtained experimental data is considered satisfactory. Furthermore, Figure 9b displays a photograph illustrating the structure of the pores achieved when the raw mixture was foamed using a heat carrier at a temperature of 130 °C for a shorter thermal treatment duration of 5 min. The scale bar at the bottom of mage indicates 1 mm divisions.

These visualizations confirm the model’s capacity to accurately predict the resulting porous structure based on specific, kinetically controlled thermal regimes.

The mean pore size was accurately predicted prior to the commencement of the experiments by meticulously setting the composition and content of the initial mixture (Table 6), the thermal treatment temperature, and the heating gas velocity within the optimal operational windows derived directly from the mathematical model. This successful validation confirms the high predictive capacity and technological reliability of the numerical model developed in this study.

As previously discussed, the thermal conductivity (λ) of PMs is directly dependent on both porosity and pore size. Specifically, the thermal conductivity coefficient is expected to increase with increasing density (ρ) and decrease with an increase in the average pore diameter (*d*). The dependence of the thermal conductivity coefficient on the material’s density and the average pore diameter can be empirically described by the following relationships:

(35) $$ln\lambda =\rho \cdot ln\left[k\cdot m\right];\;ln\lambda =2R\cdot ln\left[c\cdot Ϛ\right]$$

**Figure 10:**
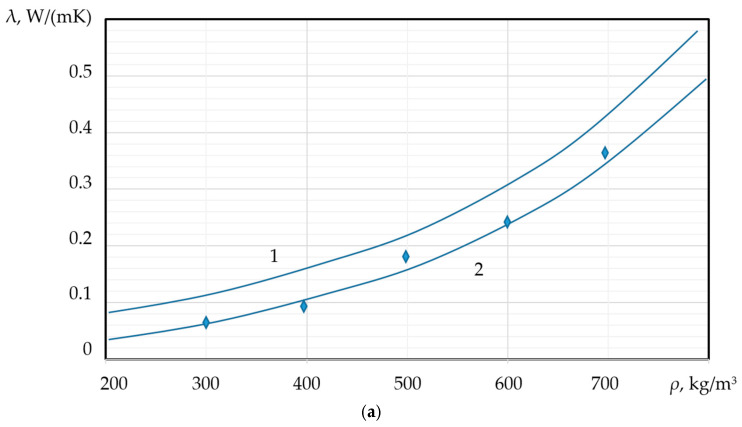

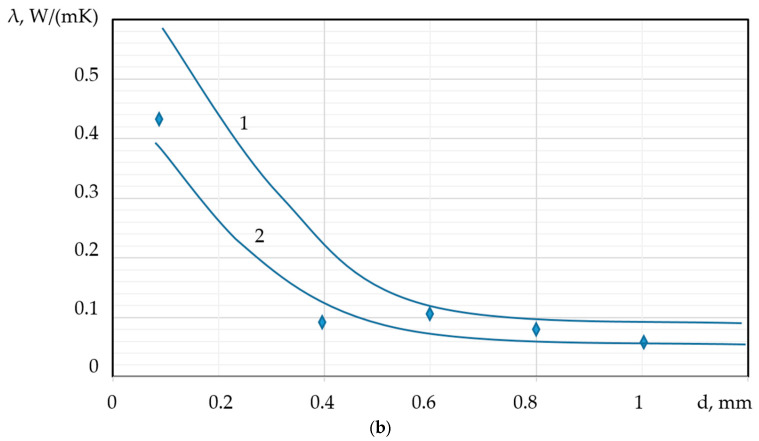
Dependence of the thermal conductivity coefficient λ  on: (**a**) density (ρ);  (**b**) average pore diameter; 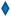 experimental data; 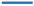 calculated values; 1—foamed concrete, 2—foamed granules (new PM).

These relationships are quantitatively demonstrated in Figure 10a,b, which illustrates the inverse correlation between λ and *d*, and the direct correlation between λ  and ρ.

## 5. Conclusions

Traditional approaches for fabricating porous materials with specific structural properties often concentrate on identifying the optimal mix of raw materials. Aligning with this established strategy, our research utilizes a mixture primarily based on technogenic components, specifically fly ash. However, this study significantly advances the field by demonstrating that the resulting porosity structure and average pore size are highly dependent on the temperature and intensity of heat exchange during the thermal treatment phase.

The principal finding is the demonstration that the resulting porosity structure and average pore size are critically dependent on the temperature and intensity of heat exchange during the thermal treatment phase, rather than solely on the starting mixture. Our developed method for producing porous material, which couples a unique raw material composition (incorporating fly ash, clay, and alkaline activators) with a novel controllable pore-forming strategy, has yielded several key scientific and technological achievements:

- the optimal thermal treatment temperatures necessary for the rapid expansion of the fly ash-based mixture were precisely determined;
- a direct, quantifiable relationship was established between the final average pore size and the duration of the intense thermal processing;
- the feasibility of controlled synthesis of lightweight porous geopolymers was definitively demonstrated by precisely adjusting the processing regimes, intensity, and duration of the heat application.

This research provides a framework for manufacturing high-performance, lightweight porous material. By enabling predictive control over microstructural features based on processing kinetics, leveraging the vortex reactor’s capabilities for rapid and intense heat transfer.

## Figures and Tables

**Table 1:** Chemical composition of raw fly ash [46].

Components	Composition (wt%) of CFA
SiO_2_	49.73
Al_2_O_3_	24.57
Fe_2_O_3_	7.15
CaO	4.65
MgO	3.19
Na_2_O	1.39
K_2_O	2.86
TiO_2_	1.12
P_2_O_5_	0.49
MnO	0.11
Loss On Ignition	7.0
SO_3_	0.76
Free CaO	0.69
ph, t = 22 °C	11.75

**Table 2:** Composition of the Raw Mixture Based on CFA.

Component	Content, Parts by Mass
CFA	100
mineral filler	up to 20
clay	up to 10
sodium hydroxide, NaOH	up to 5
$\mathrm{sodium}\;\mathrm{carbonate},\;{Na}_{2}CO_{3}$	up to 5
water	60
$\mathrm{sodium}\;\mathrm{sulfate},\;{Na}_{2}SO_{4}$	up to 5
OPC	up to 10

**Table 3:** Composition of the raw mixtures used for thermal analysis and synthesis.

Mixture Designation	CFA, Mass Parts	OPC, Mass Parts	Total Mass, Parts	OPC Content, wt%
mixture 1	80	5	85	5.9
mixture 2	70	15	85	17.6
mixture 3	50	30	80	37.5

**Table 4:** Critical temperature stages and associated physico-chemical transformations during the controlled synthesis of PMs.

Stage Designation	Temperature Range (T), °C	Physico-Chemical Process
stage I	100–120	transition from solid mixture to pseudo-plastic state (paste formation).
stage II	120–150	release of free and adsorbed water; initial pore formation (evaporation)
stage III	>150	cessation of major water loss and structure rearrangement (hardening/crystallization).

**Table 5:** Characteristics of the developed PM compared to foamed concrete.

Parameter	Unit	Foamed Granules (New PM)	Foamed Concrete	Expanded Clay Aggregate
thermal conductivity,	$\frac{W}{mK}$	0.045	0.09–0.38	0.09–0.1
compressive strength	MPa	0.6–3.0	1–5	1–3
water absorption	%	3.0	10–16	15–25
bulk density	kg/m^3^	160–180	-	-
true density	kg/m^3^	360–440	400	280–700
porosity	%	75–80	80	80
expansion coefficient		7–8	0.34–0.57	-
average pore diameter	mm	0.1–2.0	-	-

**Table 6:** Composition of the raw material mixture used for the production of porous material.

Component	Content, Parts by Mass
fly ash	100
mineral filler	up to 20
clay	up to 10
sodium hydroxide, NaOH	up to 5
$\mathrm{sodium}\;\mathrm{carbonate},\;{Na}_{2}CO_{3}$	up to 5
water	60
$\mathrm{sodium}\;\mathrm{sulfate},\;{Na}_{2}SO_{4}$	up to 5
portland cement	up to 10

**Figure A1:**
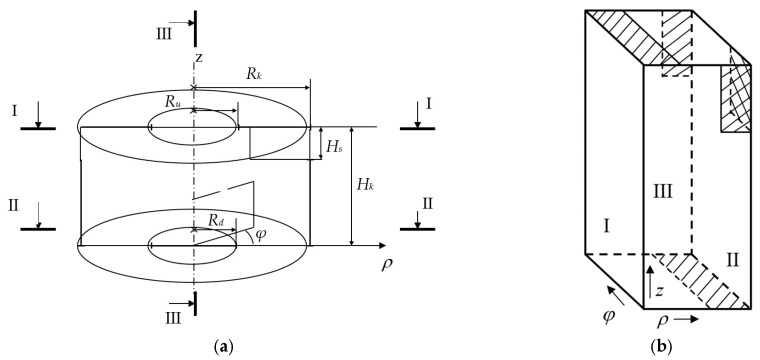
Computational domain definition for the VLR hydrodynamic model: (**a**) Axial cross-section of the computational domain for the vortex reactor: $R_{k}$ is a reactor radius, $H_{k}\;is\;the\;$ reactor height, $R_{u}$ and $R_{d}$—radii of the vertical gas flow streams at the top and bottom of the chamber, respectively, and $H_{k}\;is$ the height of the tangential gas inlet channel. Regions I, II, III delineate the boundaries for applying boundary conditions; (**b**) Transformed computational domain with ρ, φ, and z as Cartesian coordinates. Shaded regions indicate open boundaries where free-flow conditions are applied.

**Figure A2:**
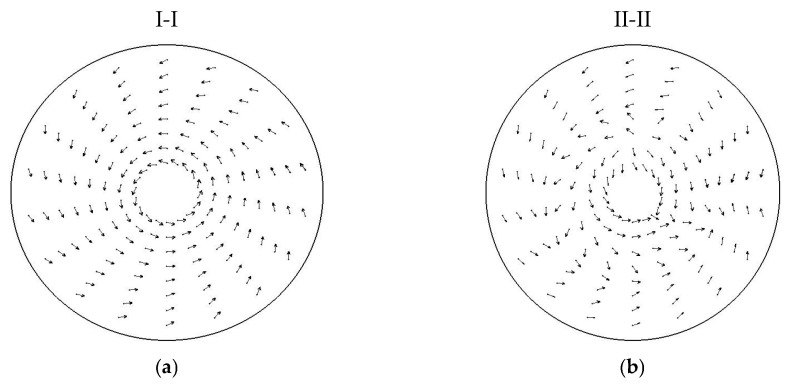
Calculated velocity direction fields of the gas in distinct horizontal cross-sections of the VLR This is a figure. Schemes follow another format. If there are multiple panels, they should be listed as: (**a**) at the level of the tangential gas inlet; (**b**) at the mid-height of the reactor.

**Figure A3:**
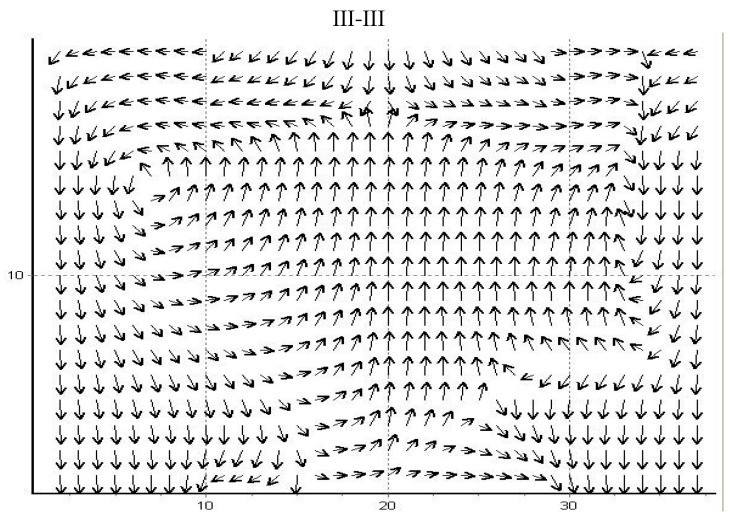
Velocity projection direction fields in the axial cross-section of the VLR. III-III represent specific cross-sectional planes used to analyze the flow profiles as shown in Figure A1a.

## Data Availability

The original contributions presented in this study are included in the article. Further inquiries can be directed to the corresponding author.

## Appendix A. Technological Implementation and VLR Modeling

### Appendix A.1. Mathematical Model of Porous Material Thermal Treatment in the Vortex Gas Layer

The kinetic analysis of pore formation highlighted the necessity of an intensive and controlled thermal treatment within an extremely narrow time window. This requirement necessitates the use of specialized high-efficiency equipment, such as a VLR. The efficiency of heat and mass transfer processes within the VLR is determined by the geometric ratios of the device, which govern its hydrodynamic performance. To rationalize the VLR’s design and operational parameters, a three-dimensional numerical simulation was employed using the highly effective physical factor splitting method, implemented in cylindrical coordinates. This approach is necessary because, due to the gas inlet configuration, the gas flows are inherently three-dimensional and highly turbulent. The computational domain used for the simulation is presented schematically in Figure A1a. In setting the boundary conditions for the numerical scheme, it is critical to precisely ensure the conjugation condition of hydrodynamic characteristics (velocities and pressures) at the boundaries. To facilitate this, the computational domain is conveniently represented in a space where the cylindrical coordinates ρ, φ, and z are treated as Cartesian coordinates (Figure A1b).

In this transformed space, the computational domain assumes the shape of a parallelepiped (Figure A1b). Face I corresponds to r=0 and degenerates into the axial line in the actual physical domain (as shown in Figure A1a, the original axial cross-section), faces II and III correspond to φ=0 and φ=2π, respectively, and must therefore be identified (coupled) to satisfy the periodicity condition of the tangential flow. The shaded regions in Figure A1b correspond to areas of free gas passage (inlet/outlet), where free-flow boundary conditions are applied. Faces II and III are linked by conjugation (coupling) conditions, while non-penetration (no-slip) boundary conditions are applied to all other solid wall boundaries. For implementation on a staggered grid, the computational domain is surrounded on all sides by a layer of ghost cells to simplify the application of boundary conditions. The conjugation conditions on faces II and III are realized by setting the ghost cell values of velocities and pressures on one face equal to the near-boundary values on the opposite face, and vice versa. The following numerical values were adopted for the geometric parameters in the simulation: $R_{k}=400\;mm$, $H_{k}=400\;mm$, $R_{u}=200\;mm$, $R_{d}=200\;mm$, and $H_{s}=200\;mm$. The method of visualizing hydrodynamic parameters in various cross-sections, by projecting the calculated velocity vectors onto them, was used to gain a comprehensive representation of the overall medium motion.

It is assumed that, given the relatively low gas velocities (significantly below the speed of sound), the gas can be accurately treated as incompressible. Furthermore, the gas flow throughout the reactor volume is presumed to be significantly turbulized, where the generation of turbulent eddies is governed by the velocity gradient perpendicular to the flow, and their transport by the flow velocity itself.

*Reynolds equations for gas dynamics.* The gas dynamics are described by the Reynolds-averaged Navier–Stokes equation (momentum conservation), which must be supplemented by the solenoidality condition derived from the continuity equation for an incompressible fluid:

(A1) $$\frac{\partial V}{\partial T}+\left(V\cdot \nabla \right)V=-\nabla \overset{´}{P+}{\nu_{ef}\nabla }^{2}V,$$

(A2) ∇V=0,  

where V is the velocity vector averaged over turbulent pulsations ms is the dynamic pressure component divided by gas density; and $\nu_{ef}$ is the effective kinematic viscosity m2s, accounting for the turbulent nature of the motion.

*Algebraic model of turbulence.* The effective kinematic viscosity, $\nu_{ef}$, is determined using an algebraic model of turbulence:

(A3) $$\nu_{ef}=\nu_{g}+\nu_{T}=\nu_{g}+l^{2}\sqrt{\frac{G}{k_{R}}},$$

where $\nu_{g}\;$ is the molecular kinematic viscosity; l and $k_{R}\;$ are length scale parameters defining the scale of turbulent mixing and the turbulent Reynolds number, respectively; and G is the localized flow velocity gradient. The turbulent mixing length, l, is empirically set equal to the computational grid step ∆, and the turbulent Reynolds number, $k_{R}$, is set to 2 to ensure the maximum adequacy of the algebraic model [51].

*Numerical solution method.* The coupled system of Equations (A1) and (A2) is solved using the physical factor splitting method over each time step ∆τ. This scheme proceeds in three stages.

Stage I. Momentum prediction explicit calculation of intermediate velocity $\overset{ˇ}{V}:$

(A4) $$\frac{\overset{ˇ}{V}-V^{n}}{∆\tau }=-\left(V^{n}\cdot \nabla \right)V^{n}+{\nu_{ef}\nabla }^{2}V^{n}.$$

This stage computes an auxiliary velocity field $\overset{ˇ}{V}$ that accurately describes the vortex motion but does not satisfy the solenoidality condition (A2).

Stage II. Pressure correction implicit calculation of pressure $P^{n+1}$. The pressure field is calculated from the condition of exact fulfillment of the solenoidality condition (A2) for the gas velocities at the n+1 time layer:

(A5) $$\frac{\partial }{\partial T}\left(\nabla \cdot V^{n+1}\right)=\nabla \left(\nabla \cdot P^{n+1}\right),$$

Stage III. Final velocity correction explicit calculation of $V^{n+1}$.

The final velocity field $V^{n+1}$ is corrected using the calculated pressure gradient:

(A6) $$\frac{V^{n+1}-\overset{ˇ}{V}}{∆\tau }=-\nabla {\overset{´}{P}}^{n+1}.$$

The spatial derivatives in the component form (e.g., radial, tangential, and axial) are approximated using a standard staggered grid finite difference scheme. The resulting Poisson equation for pressure (stage II) is solved using an iterative method.

### Appendix A.2. Hydrodynamic Flow Field Analysis

The application of the numerical method, as detailed in Equations (A1)–(A6), yields comprehensive three-dimensional data on the gas flow characteristics within the VLR. A clear understanding of the velocity and pressure fields is paramount, as they directly influence the heat transfer rate and, critically, the residence time $\tau_{res}\;$ of the particles.

Figure A2 presents the calculated velocity direction fields of the gas in two distinct horizontal cross-sections of the VLR, illustrating the evolution of the flow structure along the reactor’s height.

The flow field analysis reveals a necessary and controlled hydrodynamic transition essential for the thermal treatment process. In the upper section, at the tangential inlet level (I-I) (Figure A2a), the forced action of the supplied gas creates a powerful, well-defined toroidal vortex. This intense rotational motion is critical, as it maximizes the relative velocity between the gas and the particles, thereby achieving the highest possible initial heat transfer intensity required to trigger the rapid pore-forming reaction (as discussed in Section 3.4). However, as the gas descends to the mid-height (II-II) (Figure A2b), the rotational energy gradually dissipates due to wall friction and internal viscous forces. Consequently, the powerful vortex structure begins to deform and weaken. This controlled structural decay is crucial because the subsequent deceleration of the particles determines their residence time $\tau_{res}\;$ in the reaction zone, ensuring the material solidifies only after reaching the desired pore size.

The nature of the overall hydrodynamic pattern in the axial cross-section of the apparatus is further elucidated by Figure A3.

This axial view (Figure A3) definitively illustrates the direct consequences of the global vortex structure on the internal pressure distribution within the reactor. The intense rotational flow is shown to generate a significant low-pressure region adjacent to the upper opening. This negative pressure gradient consequently induces the ingress of ambient air into the apparatus, which represents an uncontrolled mass flow that must be accounted for in the overall system balance. Conversely, the velocity vectors in the lower section of the reactor, specifically near the particle feeder, confirm that the gas predominantly discharges from the system at this boundary. This complex, calculated three-dimensional pressure and velocity field confirms the highly turbulent nature of the flow and is paramount for accurately modeling the coupled particle-gas interaction necessary to determine the thermal treatment efficiency and overall energy balance.

### Appendix A.3. Mathematical Model of Particle Trajectory and Thermal Coupling

The thermal treatment of the raw material occurs during the period of its levitation (suspension) within the VLR, resulting from its intricate interaction with the hot gas streams. Direct experimental investigation of particle motion in such vortex devices is severely complicated by the non-linear and unsteady nature of the process. Consequently, the characteristics of the particle trajectories, which are crucial for determining the residence time $\tau_{res}$, are determined through numerical simulation utilizing the previously calculated three-dimensional gas velocity fields.

The resulting thermal treatment duration must be precisely coordinated with the kinetically required contact time (as established in Section 3.4). While an excessively long heating period will not inherently damage the material structure, a duration that is too short will result in a porous structure with characteristics below the design specifications. A key feature of this model is the incorporation of the dynamic change in particle diameter during the drying and pore-forming processes (Figure 4), which significantly affects the aerodynamic interaction with the gas phase. Therefore, the particle temperature must be calculated simultaneously with its trajectory.

Diverging from previous simplified or two-dimensional studies [51,52,53], this work computes particle motion within the full three-dimensional velocity field (Figure A2), incorporating the time-varying particle size, density, and mass, consistent with the treatment conditions defined in Section 2. We assume the particle is initially introduced falling vertically with an initial velocity $v_{p,0}$.

### Appendix A.4. Equation of Particle Motion

The total force F acting on a particle suspended in the reactor is composed of the Archimedes force and the drag force:

(A7) $$F=F_{Arcimedes}+F_{Drag}.$$

The Archimedes force is defined as follows:

(A8) $$F_{Arcimedes}=\left(\rho_{p}-\rho_{g}\right)V_{p}g.\;\;\;$$

where $V_{p}\;$ is the particle volume, $\rho_{p},\;\rho_{g}\;$ are the densities of the particle and the displaced gas, respectively. It is noted that when the particle density $\rho_{p}$ significantly exceeds the gas density $\rho_{g}$, the Archimedes force essentially reduces to the gravitational force $F_{Gravity}$.

The drag force $F_{Drag}$ is as follows:

(A9) $$F_{Drag}=\frac{1}{2}C_{R}S\rho_{g}\left(v_{g}-v_{p}\right)\left|v_{g}-v_{p}\right|.$$

where $C_{R}\;$ is the drag coefficient, S is the particle cross-section, $v_{g},\;v_{p}$ are the particle and gas velocities, respectively.

The overall equation of particle motion, incorporating the effect of added mass $m_{p,added}\;$ is expressed as follows:

(A10) $$\frac{dv_{p}}{d\tau }=\frac{F}{m_{p}}+\frac{m_{p,added}}{m_{p}}\frac{d\left(v_{g}-v_{p}\right)}{d\tau }.$$

The trajectory is calculated in cylindrical coordinates ρ, φ, z where the component form of the motion Equation (A10) is as follows:

(A11) $$\frac{dv_{\rho ,p}}{d\tau }=\frac{v_{\varphi ,p}^{2}}{\rho }+f_{\rho },$$

(A12) $$\frac{dv_{\varphi ,p}}{d\tau }=-\frac{v_{\rho ,p}v_{\varphi ,p}}{\rho }+f_{\varphi },$$

(A13) $$\frac{dv_{z,p}}{d\tau }=f_{z},$$

where the position is updated using the velocity components:

(A14) $$v_{p}=\frac{d\rho }{d\tau },\;\;\;\;\;v_{\varphi }=\rho \frac{d\varphi }{d\tau },\;\;\;v_{z}=\frac{dz}{d\tau }\;\;.\;$$

The system of Equations (A11)–(A13) is solved numerically using the explicit finite difference scheme Kramer-Euler method. The particle cross-sectional area S required for the drag force calculation is continuously updated based on the dynamically changing particle diameter d.

### Appendix A.5. Thermal Model of the Particle

The heating process, assuming particle sphericity, is governed by the one-dimensional diffusion heat transfer equation:

(A15) $$\frac{\partial T}{\partial \tau }=\alpha \left(\frac{\partial^{2}T}{\partial r^{2}}+\frac{2}{r}\frac{\partial T}{\partial r}\right),$$

where T is temperature, α is the thermal diffusivity coefficient of the particle, and r is the radial coordinate.

The boundary condition at the particle surface $r=\frac{d}{2}$ is defined by convective heat transfer:

(A16) q=−λp∂T∂rr=d2=αTs−Tg,

where q is the heat flux density, $T_{s},T_{g}\;$ are the particle surface and surrounding gas temperatures, respectively, α is the heat transfer coefficient. The coefficient α is expressed using the dimensionless Nusselt number.

(A17) α=Nu·λg,efd,

where $\lambda_{g,ef}$ is the effective thermal conductivity of the gas, which accounts for the turbulent nature of the flow, and is determined by the relation

(A18) $$\lambda_{g,ef}=C_{p,g}\rho_{g}v_{ef},$$

where $C_{p,g}$ and $\rho_{g}\;$ are the specific heat and density of the gas, and $v_{ef}\;$ is the effective kinematic viscosity, derived from the three-parameter algebraic turbulence model.

The particle temperature is calculated using the following explicit finite difference scheme:

(A19) $$T_{i}^{n+1}=T_{i}^{n}+\frac{\alpha ∆\tau }{{\left(∆r\right)}^{2}}\left[T_{i+1}^{n}-2T_{i}^{n}+T_{i-1}^{n}\right]+\frac{2\alpha ∆\tau }{r_{i}∆r}\left[T_{i+1}^{n}-T_{i}^{n}\right],$$

where ∆τ, ∆r are the time and radius steps, respectively, n is the time layer number, and i is the spatial cell number (with temperatures calculated at the cell centers). The average temperature across all cells is used to determine the dynamically changing particle diameter, thus fully coupling the thermal and hydrodynamic models.

## Appendix B. Trajectory Dependence on Injection Strategy and Flow Velocity

The results derived from the comprehensive two-phase numerical model (encompassing the gas flow analysis in Appendix A.2 and the particle trajectory model in s Appendix A.3 confirm the fundamental working hypothesis: the required particle residence time $\tau_{res}\;$ is a complex and highly controllable function of the gas flow parameters and the particle injection strategy. To quantitatively assess this relationship, a series of test simulations were conducted across varying gas flow velocities (5, 10, and 15 m/s) utilizing the three-dimensional velocity fields established in Appendix A.2.

The analysis of calculated particle trajectories (Figure A4) revealed critical design and operational dependencies.

As the injection point is moved along the axial centerline away from the tangential gas inlet towards the center of the apparatus, the particle residence time $\tau_{res}$ is drastically reduced (Figure A4a,b). This phenomenon is a direct consequence of the rapid dissipation of rotational kinetic energy in the reactor’s central core (Figure A4b). In this central zone, the capacity of the flow to generate sufficient centrifugal drag and lift forces to suspend the particles against gravity is minimal, thus accelerating the particle’s axial movement and exit. At a fixed injection location, a lower gas velocity (e.g., $v_{g}=5\frac{m}{s}$) results in the particle exiting the reactor faster, leading to a shorter thermal treatment. This counter-intuitive finding highlights the non-linear dynamics of the VLR: the centrifugal drag and lift forces generated by the vortex at lower velocities are insufficient to overcome gravity and the inherent axial slip velocity, necessitating a minimum flow intensity to ensure effective particle levitation. This finding underscores the necessity of employing a full 3D simulation approach over simplified 1D models.

Moving the injection point tangentially away from the inlet port towards the opposite wall results in a significant shortening of the trajectory and reduced $\tau_{res}$. This is attributable to the uneven distribution of the angular momentum within the reactor’s circumference, confirming that particles injected into the region opposite the high-momentum inlet are less efficiently captured and entrained by the primary circulating flow (Figure A4c,d).

The total duration of thermal processing is therefore fundamentally governed by the intensity and duration of particle scouring by the heating medium, which is complexly modulated by the initial injection conditions.
